# Participatory Methodologies, Advanced Old Age, and Possibilities for Theoretical Development

**DOI:** 10.1093/geroni/igaf122.2757

**Published:** 2025-12-31

**Authors:** Elaine Eliopoulos

**Affiliations:** Committee for Public Counsel Services- Private Panel Member, Weston, Massachusetts, United States

## Abstract

Participatory research methodologies have received increased attention with older people but remain underutilized in research with those in advanced old age. These innovative methodologies involve a participants’ collaborative co-design or co-production with the researcher. Photo-elicitation is one form of collaborative approach that enables the participant to engage with the research question through a photograph provided to them or self-initiated. This paper reports findings from a photo-elicitation study where 23 participants on the San Juan Islands of the Pacific Northwest, aged 80+, were provided digital cameras. The participants were asked to take photos that demonstrated the role their bodies played in their ability to remain social included in their communities. Their photos served as the basis for a semi structured interview which participants discussed their photographs. Participants’ narratives reflected a focus on their needs and interests despite a wide range of corporeal challenges. Their focus on community and belonging, rather than bodily challenges suggests that the photo taking facilitated capture of the nuances of their daily lives beyond the physical. These findings present possibilities for re-imagining advanced old age beyond the corporeal dominant lens and looking beyond a dominant decline model of aging. Photo-elicitation with those in advanced old age may enhance our understanding of the lived experience in the ninth decade and provide the basis for theoretical development in new directions. Its’ possibilities for informing and developing policy are promising.

